# Unlocking the hidden anatomy: synchrotron micro-tomography of the stomach, midgut, and organs in *Penaeus vannamei* and the potential route of *Enterocytozoon hepatopaenaei* (EHP) infection

**DOI:** 10.1007/s00441-026-04067-4

**Published:** 2026-04-24

**Authors:** Thanapong Kruangkum, Phakkhananan Pakawanit, Kornchanok Jaiboon, Piyachat Sanguanrut, Sukanya Saedan, Kallaya Sritunyalucksana, Siripong Thitamadee, Rapeepun Vanichviriyakit

**Affiliations:** 1 Department of Anatomy, Faculty of Science, Mahidol University, Rama VI rd., Bangkok, 10400 Thailand; 2 Center of Excellence for Shrimp Molecular Biology and Biotechnology (Centex Shrimp), Faculty of Science, Mahidol University, Rama VI Rd., Bangkok, 10400 Thailand; 3 Synchrotron Light Research Institute (Public Organization), Nakhon Ratchasima, 30000 Thailand; 4 Aquatic Animal Health Research Team, Integrative Aquaculture Biotechnology Research Group, National Center for Genetic Engineering and Biotechnology (BIOTEC), National Science and Technology Development Agency (NSTDA), Yothi office, Rama VI Rd., Bangkok, 10400 Thailand; 5 Department of Biotechnology, Faculty of Science, Mahidol University, Rama VI rd., Bangkok, 10400 Thailand; 6 Analytical Sciences and National Doping Test Institute, Mahidol University, Rama VI rd., Bangkok, 10400 Thailand

**Keywords:** Tomography, Digestive tract, *Penaeus vannamei*, Gastric sieve, Stomach-midgut junction

## Abstract

Due to the structural complexity of the crustacean digestive system, this study characterizes the three-dimensional (3D) morphology and histology of the juvenile *Penaeus vannamei* alimentary tract using synchrotron radiation X-ray tomographic microscopy (SR-XTM) and H&E staining. SR-XTM volumetric imaging effectively reveals the structural interconnections among the pyloric stomach (PS), hepatopancreas (HP), and midgut (MG). Histological analysis further distinguishes the regional processing of fine and coarse chyme, which are segregated by a pair of lateral pyloric valves (LPVs). These valves function as mechanical barriers to prevent mixing of coarse and fine chyme within the anterior MG. Furthermore, this study identifies a secondary communication pathway, a sub-LPV channel, that enables a direct connection between the MG and the HP. In situ hybridization of EHP-specific transcripts identifies significant *Enterocytozoon hepatopenaei* (EHP) infection in cells lining the primary hepatopancreatic chamber and proximal regions of the secondary and tertiary HP tubules. Given that the gastric sieve restricts pathogen transmission by particle size, our findings suggest the sub-LPV channel serves as a critical alternative route for the oral transmission of EHP spores. This study provides a foundational structural framework for understanding shrimp digestive anatomy and host–pathogen internalization.

**Supplementary Information:**

The online version contains supplementary material available at 10.1007/s00441-026-04067-4.

## Introduction

The alimentary tract is vital for the survival of shrimp and other crustaceans, playing a key role in ingestion, digestion, and nutrient absorption, all of which are essential for growth. Additionally, a disease such as hepatopancreatic microsporidiosis (HPM), caused by the microsporidian parasite *Enterocytozoon hepatopenaei* (EHP), is suspected to be associated with and transmitted through this pathway in shrimp cultures (Tang et al. 2016; Chaijarasphong et al. 2021). The possible route of infection, including the mechanism by which it reaches the target organs, remains unclear (Chaijarasphong et al. 2021). Understanding the shrimp alimentary tract could provide greater insight into potential infection mechanisms. The interrelationships among organs in the alimentary tract of penaeid shrimp have primarily been studied using histological and morphological approaches (Young and Joseph 1959; Caceci et al. 1988; Ceccaldi 1989; Pattarayingsakul et al. 2019; Bell and Lightner 1988). The alimentary tract is often studied using fragmented tissue pieces, which limits our understanding of its whole organization. Its complex internal structures make comprehension challenging. Advanced investigations into gastric anatomy and its structural connections are essential for gaining a deeper insight.

Micro-computed tomography (µ-CT) is widely used in biomaterials research to visualize internal morphology non-invasively through 3D reconstruction from stacked images. It has been applied across various organisms (Metscher 2009), including 3D phenotypic screening and histopathological analysis in zebrafish (Ding et al. 2019). In invertebrates, it has helped examine the micro-organization of the insect brain and the micro-organization of the host-parasite ant (Martín-Vega et al. 2018). Synchrotron radiation X-ray tomographic microscopy (SR-XTM) further enhances 3D visualization by reconstructing structures from serial optical sections at a synchrotron beamline, which could be essential for this approach rather than scanning electron microscopy (SEM) (Caceci et al. 1988).

The anatomical and morphological organization of the alimentary tract in white shrimp, *Penaeus vannamei*, particularly organ junctions, remains unclear. Routine H&E staining provides limited insight into organ interactions, and the valvular system of the gastric wall remains ambiguous (Ceccaldi 1989). This knowledge gap hinders understanding of ingestion, digestion, food transport (Kasamechotchung et al. 2025), and pathogen transmission. Our recent study identified the gastric sieve in the posterior stomach as a key structure for selecting crude food particles (Pattarayingsakul et al. 2019). Situated near the posterior stomach-midgut junction—adjacent to the hepatopancreatic opening—this structure serves as a potential reference point for further investigation. However, many aspects remain unclear and warrant further study.

This study aimed to investigate the structures at the junction of the posterior stomach and anterior midgut, as well as the associated organs, using the constructed SR-XTM imaging analyses. Moreover, this data would be considered alongside the histology and morphology of the structures using routine staining and scanning electron microscopy. This is the first report to use µ-CT image reconstruction with synchrotron radiation to illustrate the structures within the alimentary tract of the white-leg shrimp, aligning with those observed in routinely histologically stained tissues. This study would provide valuable guidance by illustrating the organization of organs at the junction of the posterior stomach, midgut, and hepatopancreas in shrimp and proposing a potential pathogen entry route into the hepatopancreas.

## Materials and methods

### Animal ethics

The animals and tissue samples used in this study were handled under supervision and in accordance with the Thai national guidelines on the care and use of animals for scientific purposes, as per permit MUSC64‒035‒584 from the Institutional Care and Use Committee, Faculty of Science, Mahidol University.

### Animal use and tissue collections

Two grams of juvenile shrimps (*Penaeus vannamei*) was used as an animal model to demonstrate the similarities and differences in the organization of the alimentary tract, especially at the portion of the posterior stomach (PS), midgut proper (MG), and the extrusion of associated organs, including the anterior midgut cecum (AMC) and hepatopancreas (HP). The shrimps were euthanized by being immersed in an ice basket before undergoing further procedures. For routine H&E staining, the shrimps were injected with Davidson’s fixative solution in several regions around the cephalothorax to prevent autolysis, while avoiding damage to important internal structures, such as the gastrointestinal tract. The shrimps were cut at the thorax-abdominal junction before being immersed in the fixative solution for 24 h at room temperature. In addition, the specimens for X-ray µ-CT and scanning electron microscopy (SEM) were prepared using the same procedure, but with a fixative containing 2.5% glutaraldehyde and 2% paraformaldehyde instead.

### Tissue preparation for scanning electron microscopy (SEM)

The samples from the juvenile white-leg shrimps were washed in 0.1 M phosphate buffer (PB) after fixation with a glutaraldehyde-paraformaldehyde fixative solution for over five times of washing, as described in Pattarayingsakul et al. (2019). The fixed tissues were post-fixed by immersion in 1% osmium tetroxide (OsO_4_) in 0.1 M PB at 4 °C for 2 h until the samples turned black. Then, they were gently washed with drinking water for 30 min, three times. Dehydration at increasing ethanol concentrations (30% to 100%) was performed twice at 4 °C before critical-point drying. The duration of each dehydration step was around 30–40 min, while the final dehydration in absolute ethanol was performed four times, 30 min each, at 4 °C. A critical-point drying machine (Hitachi HCP-2) using liquid CO_2_ was used, and the sample was then placed and mounted on stubs with conductive carbon tape. They were consequently coated with platinum and palladium using a Hitachi E-120 ion sputter. The coated tissues were kept in a chamber containing silica gel to prevent any humidity until examination. The coated tissues were examined and photographed using a Hitachi S-2500 scanning electron microscope.

### Synchrotron radiation X-ray tomographic microscopy (SR-XTM)

**Fig. 1:**
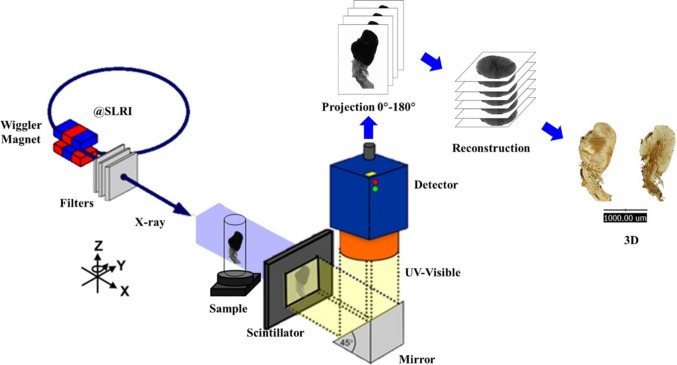
The schematic illustration shows the procedure for the sample and image analyses using synchrotron radiation X-ray tomographic microscopy (SR-XTM)

The fixed samples were prepared using the same procedures as described in the SEM technique, without metal coating. The dried tissues were carried in soft packages to the Synchrotron Light Research Institute (Public Organization), Nakhon Ratchasima, Thailand, for analysis. Synchrotron radiation X-ray tomographic microscopy (SR-XTM) was performed at beamline 1.2 W. The methodology for sample and image analyses was based on SR-XTM, as shown in Fig. 1. For a complete dataset, X-ray projections of the sample were collected over 180° with 0.1° angular increments. Polychromatic X-rays were attenuated using a 350-micron-thick aluminum foil to minimize artifacts, with a mean energy of approximately 11.5 keV. An sCMOS camera collected the X-ray projections with a pixel size of 3.61 µm. The data were pre-processed and reconstructed in three dimensions using a filtered back-projection algorithm in Octopus Reconstruction Software (Desrues et al. 2006). After that, the reconstructed images were visualized in three dimensions using Drishti software (Limaye 2012) and exported into MWV and MP4 files for image analysis and capture. The studies of reconstructed images were analyzed along the three-dimensional axes (X–Y-Z) of the shrimp’s anatomical plan. The rendered movies from reconstruction and raw data were captured as a series of single images. Those images are the best representatives for further analysis.

### Histological investigation using standard staining with H&E

The tissues fixed in Davidson’s fixative solution were rinsed multiple times in 70% ethanol. Then, they were transferred to the automatic tissue processor (Leica TP1020) for dehydration and paraffin infiltration by passing through steps with increasing ethanol concentrations (70% to 100%) before clearing with two steps of xylene and paraffin infiltration. The tissues were embedded in melted paraplast and placed on a cold plate to solidify. The tissue block was manually sliced into serial thin ribbons using a microtome (Leica RM2125RT). A 5- to 6-µm tissue section was placed in the 42 °C water bath, then transferred to glass slides and left on a warm plate at 42 °C overnight. For staining, the tissue sections were deparaffinized and rehydrated by passing through three steps of toluene solvent chambers, followed by a set of jars containing a decreasing series of ethanol concentrations (100% to 50% ethanol), 5 min each, before being stained with hematoxylin and eosin (H&E). For hematoxylin, the stained tissue slides were developed with the purple signal by immersing them in a jar of tap water for 5–10 min, then rinsing. The sectioned samples were dehydrated and cleared by placing them in jars containing increasing concentrations of ethanol (80% to 95%) for 5 min each, then two steps of 100% ethanol for 10 min each, and finally cleared with two steps in jars containing toluene, 10 min each. Finally, the samples were covered with coverslips before examination by a light microscope (Leica DM750) and photographed with a digital camera (Leica ICC50 HD). Pseudo-colors were used to label the specific areas or structures mentioned, which were created in PowerPoint’s drawing mode.

### The anatomy terms nomenclature

The anatomical nomenclature for the shrimp digestive tract follows the framework established by Roberts (1966). While modern terminology—notably proposed by Cervellione et al. (2017)—suggests “perigastric organ” as a more accurate term, “hepatopancreas” remains the more widely recognized classical term for the organ surrounding the posterior stomach. To maintain clarity and consistency with broader literature, this study adopts “hepatopancreas” as the primary term, while acknowledging “perigastric organ” as its modern equivalent.

### EHP infection and tissue collection

Healthy *P. vannamei* shrimp were cocultured with *Enterocytozoon hepatopenaei* (EHP)–infected shrimp for 7 days. After the cohabitation period, shrimp were randomly sampled for EHP detection using qPCR (Munkongwongsiri et al. 2021), while the remaining shrimp were subjected to EHP detection using in situ hybridization (ISH). The cephalothorax of EHP-infected shrimp and the uninfected ones were fixed with Davidson’s fixative for 24 h and then transferred to 70% ethanol before processing for paraffin embedding. The tissue block was sectioned at 5 µm for in situ hybridization. For qPCR analysis, shrimp HP was collected, and DNA was extracted using an Exgene™ Cell SV (GeneAll, Korea) according to the manufacturer’s protocol. The total DNA concentration was measured using a Nanodrop microvolume spectrophotometer. The DNA sample was used to detect EHP by qPCR. The 20 µL of qPCR reaction contained 10 ng of DNA template with 1 × SYBR-green master mix (Kapa Biosystems, USA), 0.2 μM each of SWP-F1 (5′-TTGCAGAGT GTTGTTAAGGGT TT-3′) and SWP-R2 primer (5′-GCTGTTTGTCTCCAACTGTATTTGA-3′) (Munkongwongsiri et al. 2021).

### In situ hybridization

The localization of microsporidia was conducted using in situ hybridization, in which specific probes were designed to bind exclusively to EHP (as published by Tangprasittipap et al. 2013). This study used a labeled probe prepared with the DIG-PCR labeling kit (Roche, Germany) specific for ssu rRNA gene probe preparation (ENF779: 5′-CAGCAGGCG CGAAAATTGTCCA-3′ and ENR779: 5′-AAGAGATATTGTATTGCGCTTGCTG-3′, amplicon size 779 bp) (Tangprasittipap et al. 2013). Histological sections of EHP-infected shrimp that passed through the stomach, hepatopancreas, and midgut were used in this experiment. The sections were predigested with 10 µg/mL Proteinase K (Sigma, Germany) in TNE buffer for 10 min at 37 °C. A pre-hybridization buffer containing 4 × SSC and 50% (v/v) deionized formamide was applied by overlaying 200 µL onto the tissues. They were incubated in a moist chamber and placed in a 37 °C incubator for 30 min. Afterward, they were replaced with an equal volume of hybridization buffer containing the DIG-labeled probe (approximately 200 ng per slide). The tissue sections were covered with a coverslip to prevent evaporation during incubation overnight. After washing with wash buffer, the sections were incubated with 0.5% blocking solution (Roche, Germany) for 30 min at room temperature. Alkaline phosphatase-conjugated anti-digoxigenin antibody (1:500 dilution) was applied to the tissues and incubated for 1 h at room temperature. Non-specific binding and unbound antibodies were washed several times. The detection buffer (100 mM Tris–HCl, 100 mM NaCl, and 50 mM MgCl_2_, pH 9.5), containing NBT-BCIP substrate (Roche, Germany), was applied before chromogenic development. The timing of color development in the tissue after probe application was compared with that of the negative controls. The negative controls in the system were divided into two conditions: First, a tissue section adjacent to the treated one from the infected shrimps was used, in which the specific probe was omitted. Second, the tissue section from an uninfected shrimp was applied with the specific probe. The nuclear counterstaining was performed using Bismarck brown Y (Sigma, USA). The tissues were examined under a light microscope (Leica DM750) and photographed with a digital camera (Leica ICC50 HD).

## Results

**Fig. 2:**
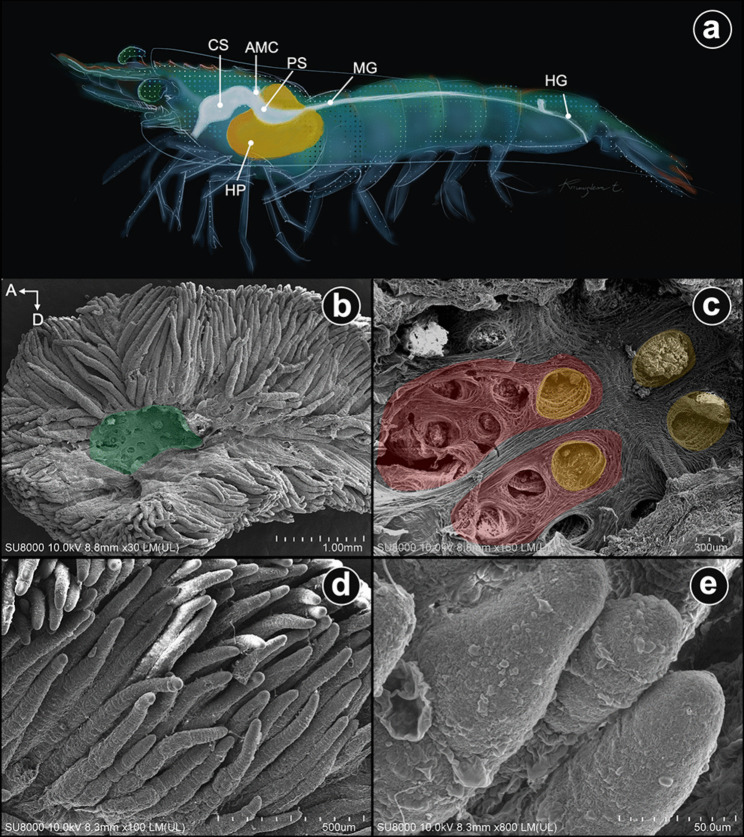
Schematic illustration of the digestive tract (**a**) and SEM images of the hepatopancreas (HP) (**b**–**e**). **a** Schematic illustration of the digestive tract of the shrimp. **b** The primary hepatopancreatic or collecting space (labeled in green). This part receives chyme from the pyloric stomach through the hepatopancreatic duct. From this primary space, chyme is distributed into a hierarchical network of secondary and tertiary ducts. **c** The secondary (HP) ducts are encircled by a large folding of fibrous bands forming the large hole (red area) containing several small tertiary ducts (yellow areas). **d** Low magnification and **e** high magnification of the external surface of the HP tubules. Abbreviations: CS, cardiac stomach; PS, pyloric or posterior stomach; AMC, anterior midgut cecum; MG, midgut; HG, hindgut (© Joel Sartore/Photo Ark as the original image referenced for the artist rendition; drawn by Kruangkum T.)

The white shrimp (*Penaeus vannamei*) digestive tract is a continuous part, extending from the mouth opening and connecting with the short esophagus. The stomach is divided into two distinguished portions, the anterior or cardiac stomach (CS) and the posterior or pyloric stomach (PS). The caudal portion of the PS is the area of interconnection of four structures: PS centrally, midgut (MG) caudally, anterior midgut ceca (AMC) dorsally, and hepatopancreas (HP) surrounding the PS and the HP ducts, ventrolaterally. The small, string-like MG extends caudally to the last abdominal segment and becomes the hindgut (HG) after passing the posterior midgut cecum (PMC) (Fig. 2a).

The medial side morphology of the left HP was revealed using SEM. It was removed from the ventroposterior part of the posterior stomach. A large portion of the primary HP space is on each side (Fig. 2b). Within the primary space, a surrounding connective tissue band forms the extensive annular system, which contains small annuli corresponding to the secondary and tertiary duct openings, respectively (Fig. 2c). Externally, the numerous bunt endings originate from individual lobules (Fig. 2d, e). Each cylindrical tubule presents annular rings that form as the worm-like segment (Fig. 2d).

**Fig. 3:**
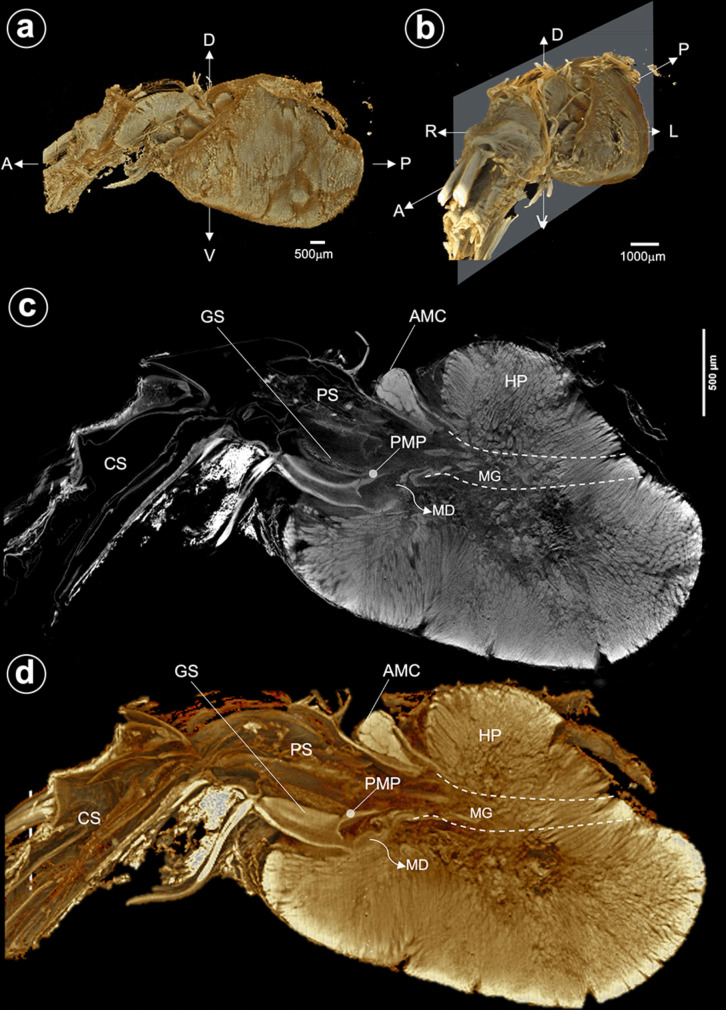
The μCT images of the stomach (St) and hepatopancreas (HP). **a** Left-side view and **b** left anterior-lateral of the St-HP of a juvenile shrimp. **c** The parasagittal section by μCT image and **d** its reconstruction presents the internal interconnection among the pyloric stomach (PS), anterior midgut cecum (AMC), HP, and midgut (MG). Abbreviation:A, anterior; P, posterior; D, dorsal; V, ventral; R, right; L, left; GS, gastric sieve; MD, main duct; PMP, posterior median plate

Three-dimensional micro (μ)-CT of the stomach-HP complex demonstrates the anatomical interrelationship of the juvenile digestive structures from the left lateral (Fig. 3a) and anterior-lateral of the left aspects (Fig. 3b). On the parasagittal plane, the internal structures of the stomach-HP complex were demonstrated using the original signal (Fig. 3c) and the modified artificial signal (Fig. 3d) from the constructed structures. The gastric sieve (GS) is in the inferior caudal part of the PS. The posterior median plate (PMP) of the GS is visible (Figs. 3c, d). The most caudal part of the PS connects to the anterior part of the MG and the opening of the HP duct (MD) inferiorly (Figs. 3c, d). Dorsal to the caudal part of PS is a junction of a bilobular knob of the AMC, located anterior to the dorsal lobe of the HP. With a modified signal, it revealed a clearer signal from a few shelves at the lateral margin of the internal wall of PS (Fig. 3d).

**Fig. 4:**
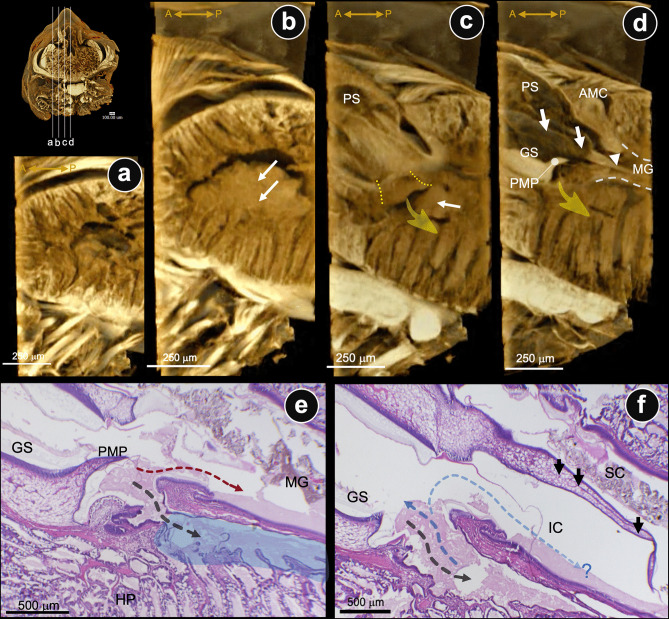
Parasagittal serial sections by μCT and H&E-staining images passing through the interconnected portion of the pyloric stomach (PS)-midgut (MG)-hepatopancreas (HP) of the juvenile shrimp. **a**–**d** Parasagittal sections from left-lateral to medial sides of the gastrointestinal structures by μCT technique revealed an accumulation of chyme in the HP spaces (white arrows in **b** and **c**), and the opening area of the main HP duct (yellow dashed lines) (yellow arrows in **c** and **d** indicated the HP space). **e**, **f** Parasagittal H&E-stained micrographs of the interconnection of three organs present the interconnection of spaces posterior to the GS and secondary duct of the posterior HP lobes (blue labelled area in **e**). **f** A right lateral ridge of PS (white arrowhead in **d** and black arrows in **f**) divided the MG lumen into superior and inferior chambers (SC and IC, respectively) for the separation of crude and fine chyme. Dashed lines are drawn to propose the direction of chyme movements. Abbreviations: A, anterior; P, posterior; PMP, posterior median plate

**Fig. 5:**
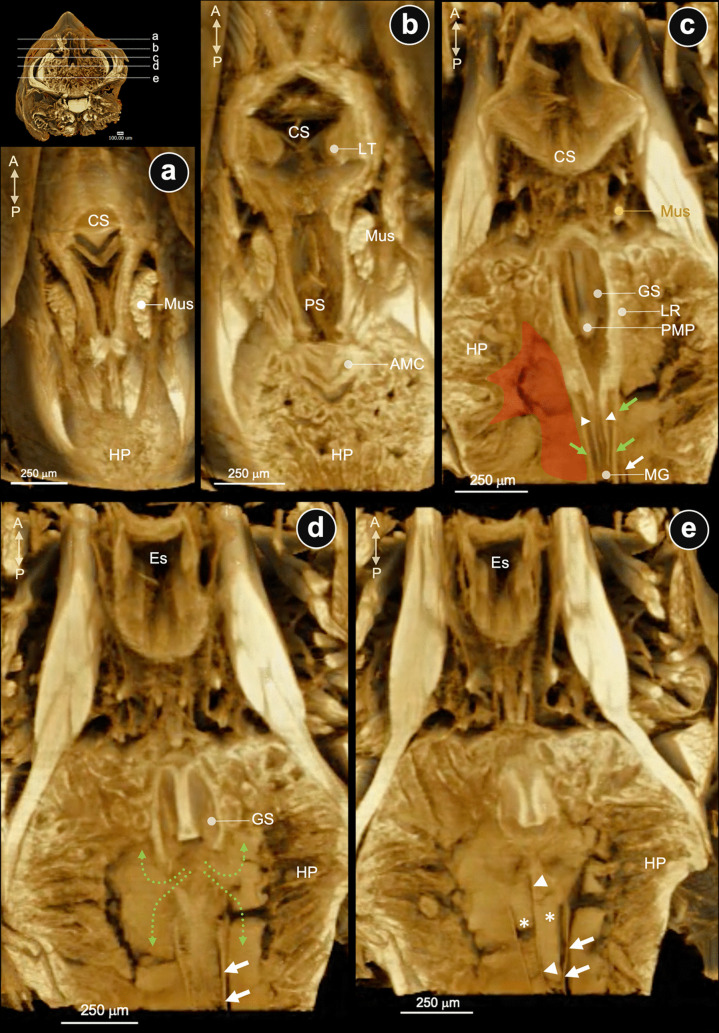
Serial horizontal section μCT images passing through the interconnected portion of the pyloric stomach-midgut-hepatopancreas of the juvenile shrimp. **a**–**e** Horizontal sections from dorsal to ventral of the pyloric stomach (PS). **c** The horizontal cut through the pyloric-midgut junction. The posterior median plate (PMP) and the lateral ridge (LR) of the gastric sieve (GS) are visible in the pyloric chamber. A pair of the lateral pyloric valves (arrowheads) is shown extending from the lateral ridge (LR) into the lumen of the midgut (MG). The lateral MG wall is indicated by the green arrows. In this plane, the hepatopancreatic (HP) space is characterized by an accumulation of fine chyme (red-labeled area), and the capsule enveloping the posterior HP lobe is visible (white arrows). **d** Ventral to the section in (**c**), the HP duct openings are visible, facilitating communication between the PS and the HP space (green-dotted arrows). An accumulation of fine chyme is evident in the HP space. **e** A more ventral section relative to the plane shown in (**d**) reveals a median septum (white arrowheads) that bisects the posterior HP lobes. Accumulations of fine chyme are indicated by asterisks. White arrows in (**d**) and (**e**) indicate the capsule enveloping the posterior HP lobe. Abbreviations: CS, cardiac stomach; LT, lateral teeth;Mus, muscle

**Fig. 6:**
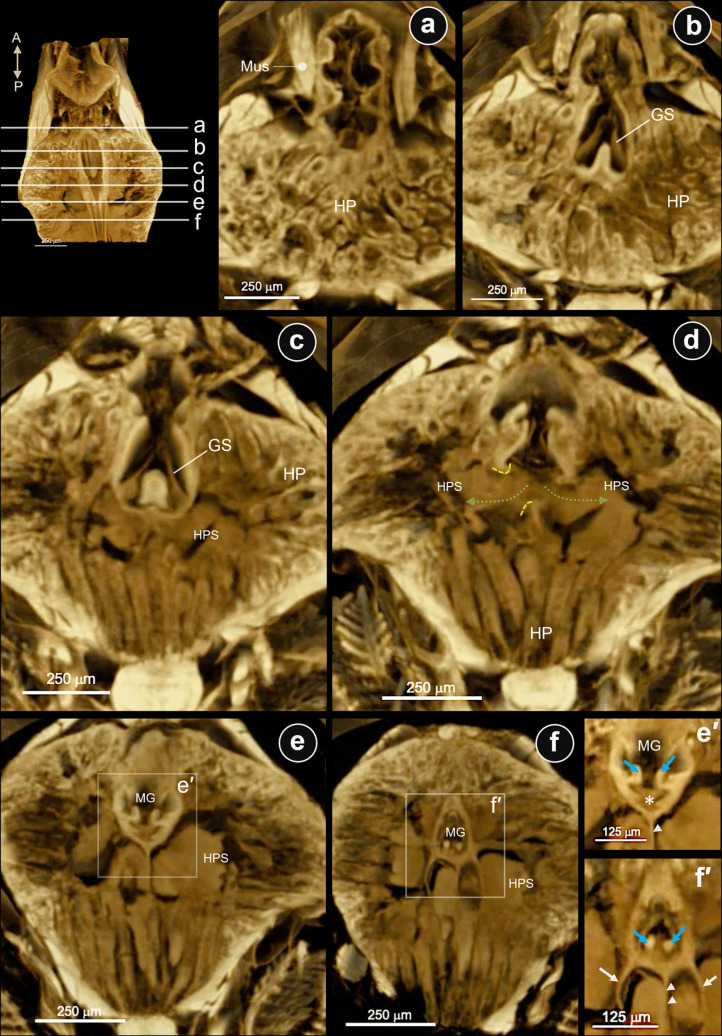
Coronal section μCT images passing through the interconnected portion of the pyloric stomach (PS)-midgut (MG)-hepatopancreas (HP) of the juvenile shrimp. **a**–**f** Coronal sections from the rostral to the caudal of the PS and MG. **d** At the cut area where the HP duct (yellow dotted line) opens to the HP space (green dotted arrow), and at the beginning of the early midgut. (e′ and f′) High magnification of coronal sections of **e** and **f**, respectively. The early (e′) and middle (f′) parts of the lateral pyloric valves project along the early midgut lumen (light blue arrows). (e′) Fine particulates of digested food were detected inside (asterisk). (f′) The lateral wall (white arrow) and the median septum (white arrowheads) of the posterior lobe of HP are visible ventral to the midgut

**Fig. 7:**
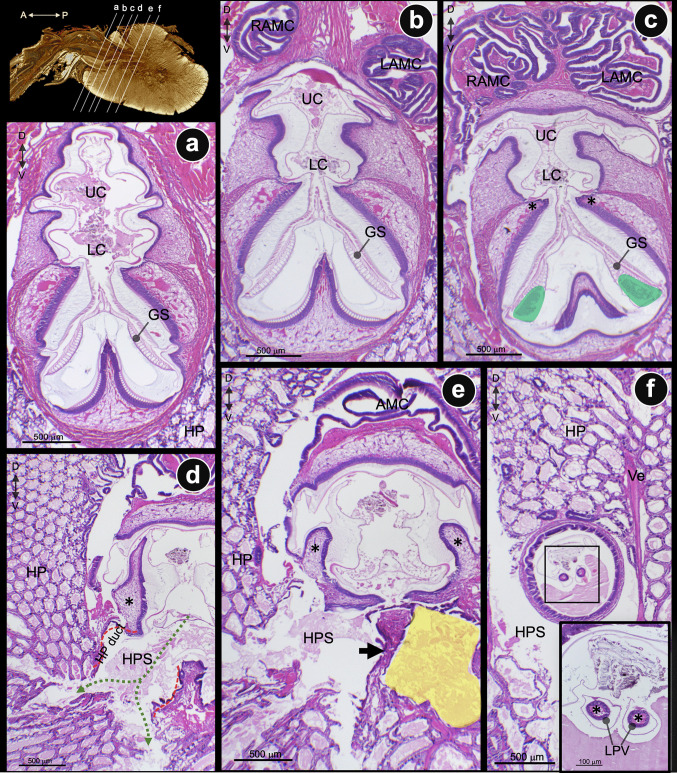
Coronal H&E-stained sections of the stomach-midgut-hepatopancreas junction. **a**–**f** A series of coronal sections arranged from rostral to caudal, illustrating the structural transition between the pyloric stomach (PS) and the midgut (MG). Green-shaded areas in **c** represent the confluence of the sub-gastric spaces, where filtered chyme accumulates prior to its entry into the main hepatopancreatic (HP) duct (indicated by red-dashed lines). Green-dashed arrows indicate the specific hepatopancreatic-stomach (HP-PS) communicating space. The asterisks (*) in **c**–**e** denote the continuous structural extension of the lateral ridge of PS into the lateral pyloric valves (LPVs). A yellow-shaded area highlights the right side of the posterior HP lobe, which is partitioned by a median septum (arrow in **e**). A pair of LPVs is visible within the MG lumen, potentially acting as a functional barrier to segregate coarse chyme dorsally and fine chyme ventrally in the MG. Abbreviations: UC, upper chamber; LC, lower chamber; RAMC, right anteriormidgut cecum; LAMC, left anterior midgut cecum; AMC, anterior midgut ceca; HPS, hepatopancreatic space; Ve, vessel

The µ-CT images have been studied in three reference axes of the cephalothorax: sagittal (Fig. 4), horizontal (Fig. 5), and coronal (Fig. 6) planes. Those of the three reference axes would be described and considered based on the location of the structures relevant to the GS. Descriptively, the stomach-hepatopancreas (St-HP) complex of the juvenile shrimps was examined on the whole cephalothorax. The parasagittal sections through the lateral part of the HP lobe showed a large cavity (Fig. 4a) fully accumulated with fine-food deposits (Fig. 4b, arrows). A large chamber in the left main HP space was observed, showing the main HP duct (Fig. 4c, yellow dashed line and arrow) opening and food accumulation in the HP space (Fig. 4c, white arrow). Close to the median half (midline) of the posterior stomach (PS), which contains the gastric sieve (GS), there was a large cavity ventrally connected to the HP duct and caudally to the midgut (MG) (Fig. 4d, yellow arrow: main HP). The posterior median plate (PMP) is situated at the caudal extremity of the GS along the midline, serving as a structural boundary between the GS and HP duct (Fig. 4d, e). The lateral margin of the PS wall presents the extrusion of the lateral ridge (LR), which is found on both lateral sides of the PS (Fig. 4d, white arrows). The caudal part of the LR of the interior wall of PS is a continuous projection of a cuticle-coated structure that extends into the lumen of the MG (Fig. 4d, white arrowhead). This structure is called the “lateral pyloric valve” in the anterior midgut. Corresponding to the routine H&E-stained tissues in the parasagittal sections of the PS-MG junction, there is the HP duct portal opening connected to the posterior lobule of HP (Fig. 4e, blue area). H&E-stained tissue revealed a continuation of fine food particles extending from behind the GS to the MG and the inferior lobule of HP (Fig. 4e, f; curved dot arrows). This indicated the route of the food-transmitted pathway among these three structures. Laterally, the caudal extension of a pair of lateral ridges of PS extended into the MG lumen. They divide the anterior midgut lumen into two sub-chambers: superior and inferior chambers (SC and IC, respectively) (Fig. 4f, black short arrows, and Fig. 7f, asterisks). This result revealed a clear distinction between food particles: the SC contained coarse particles, while the IC collected fine particles within the MG (Figs. 4f and 7f).

The horizontal serial sections from the dorsal to the ventral part of the St-HP complex revealed that the PS is distinguished from the CS by the surroundings of the HP (Fig. 5a, b). The horizontal serial sections from the dorsal to the ventral part of the St-HP complex revealed that the HP covers almost the entire area of the PS (Fig. 5a–c). Moreover, a pair of descending transverse muscles is observed at the CS-PS junction, anterior to the HP. The anterior midgut cecum (AMC) is located on the ventral margin of the superior lobe of the HP (Fig. 5b). Horizontal cuts through the upper chamber of the PS reveal a pair of LR on the lateral wall of the PS, continuously extending to the MG’s lumen (Fig. 5c, arrows). The PMP occupied the caudal-most portion of the GS (Fig. 5c). There are a pair of main HP ducts and their openings that connect to the PS and are located posterior to the GS (Fig. 5c, d). There are two hepatopancreatic openings on the left and right, located posterior to the GS, that connect the HP with the PS (Fig. 5c, d). Ventral to the early MG epithelial lining is the connection of the inferior HP lobules, laterally (Fig. 5d, e, white arrows). Moreover, the midsagittal line ventral to the early MG presents a medial septum (Fig. 5e, arrowheads, and Fig. 7e, black arrow) that separates the HP posterior lobule into two chambers (Fig. 5e, asterisks).

The coronal serial sections by the μCT images (Fig. 6) and H&E-stained tissue (Fig. 7) passing through the PS-HP complex were collected from the rostral to the caudal. A pair of external gastric muscles runs close to the lateral wall of the PS (Fig. 6a), while the gastric sieve structure is located at the base of the PS (Fig. 6b, c). The primary hepatopancreatic spaces of the left and right HP are filled with fine food particles (Fig. 6c, d). The continuity of the PS chamber and the left and right primary HP spaces is clearly demonstrated (Fig. 6d). The caudal portion of the PS has a smaller chamber diameter than the rostral portion and continues with the MG lumen (Fig. 6e, f). The LR of the PS forms a pair of lateral pyloric valves (LPV), which extend into the early midgut lumen (Fig. 6(e′, f′); blue arrows and Fig. 7c–f, asterisks). SEM photographs of the LPVs present numerous long and distal hook-like setae on their external surface (Figure S1c). A cross-section of the LPVs shows the lining epithelium covered by a thin cuticle (Figure S1d). Furthermore, the detailed histology of the PS was examined to confirm the presence of structures identified by μCT (Fig. 7). The H&E-stained images show two sub-chambers of the PS: the upper and lower chambers (Fig. 7a–c). A pair of AMCs is presented dorsally to the PS, while the GS is located at the base of the PS (Fig. 7a–c). In the caudal parts of the GS, fine food particles accumulated in a fused chamber that communicates directly with the primary hepatopancreatic space (Fig. 7c–e). In the caudal parts of the GS, the small chambers fused to form the accumulative portion or space for delivering fine food particles into the main HP duct (Fig. 7c–e).

**Fig. 8:**
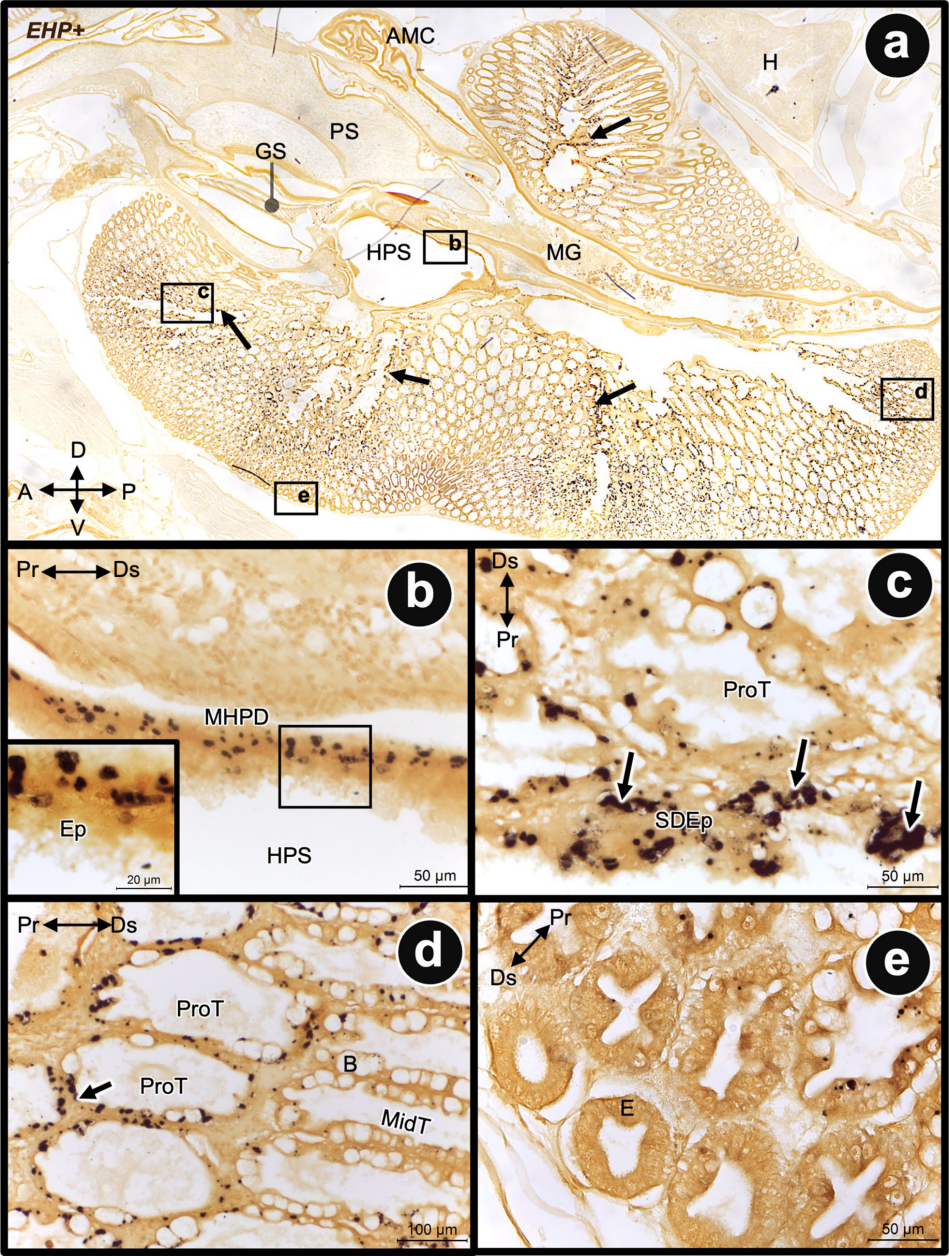
Detection of EHP by in situ hybridization in the juvenile hepatopancreas of *P. vannamei*. **a** A micrograph showing a strong EHP-positive signal in the primary and secondary ducts of HP lobules (arrows). **b**–**d** The positive signal was strongly detected in the cells lining the main hepatopancreatic duct (MHPD), secondary duct epithelium (SDEp, arrows in **c**), and the proximal zone of tertiary hepatopancreatic tubules (ProT, arrow in **d**). **e** Absence of a positive signal was found in the distal tubule, where E-cells are located. Abbreviation: ProT, proximal part of tertiary hepatopancreatic tubule; MidT, middle part of tertiary hepatopancreatic tubule; E, E-cell; B, B-cell; Ds, distal; Pr, proximal; HPS, hepatopancreatic sinus (primary hepatopancreatic duct)

To investigate the potential route of EHP infection into the HP, in situ hybridization of EHP ssu rRNA was performed on sections of shrimp cephalothorax tissue, focusing on the PS-HP-MG junction. EHP infection in the shrimp sample was confirmed by qPCR analysis (Figure S2). A positive signal for EHP was detected in high intensity in the epithelial cells lining the primary hepatopancreatic space, which continues from those lining the early MG (Fig. 8a, b). In addition, positive signals were detected in the secondary duct and the proximal region of the tertiary HP tubules, particularly in the B- and R-cells (Fig. 8c, d). In contrast, no positive EHP signal was observed in the terminal (distal) part of the HP tubule, where the E-cells are located (Figs. 8e and S3e-h).

## Discussion

This study offers the first comprehensive anatomical visualization of complications within the alimentary tract of juvenile *Penaeus vannamei*, achieved through synchrotron radiation X-ray tomographic microscopy (SR-XTM) and micro-computational tomography (µ-CT) at the junction of the pyloric stomach and midgut. This region provides important insights into the organization of various organs, including the hepatopancreas (HP) and the anterior midgut ceca (AMC). The structural analysis of the shrimp cephalothorax was conducted along three planes: sagittal, horizontal, and coronal. Moreover, standard hematoxylin and eosin (H&E) staining was performed on the tissue slices to enhance the observed details. Importantly, this study has revealed information not documented in previous conventional studies (Roberts 1966; Caceci et al. 1988; Ceccaldi 1989; Pattarayingsakul et al. 2019), providing a deeper understanding of the specific functions of the relevant structures. While we recognize that histological methods may appear “old-fashioned” in today’s research landscape, they remain essential components that can significantly contribute to a broader understanding of anatomy in future investigations. 

In this study, we employed the synergistic application of SR-XTM µ-CT, standard histology, and SEM to enhance the visualization and understanding of the organizational structure of shrimp alimentary organs. Micro-CT was selected to illustrate the digestive structures in the common spider-crab larva (*Maja brachydactyla* Balss, 1922), the organization of the nervous system of the spider (Rivera‐Quiroz and Miller 2022), and in an alpheid snapping shrimp (Chapuis et al. 2024). In the commercial shrimp species, *P. vannamei*, the morphology of the digestive organs revealed by this technique is limited. However, micro-CT has also been applied to study the brain (Meth et al. 2017) and mandible (Zhu et al. 2024) of this species. In this study, using SR-XTM µ-CT, we discovered that the posterior stomach creates shelf-like structures on both sides of the lateral wall. At the junction of the stomach, midgut, and hepatopancreas, the gastric sieve is located at the ventral median position of the intersection. The connecting channel from the posterior stomach to the hepatopancreas’s primary chamber was found to be more posterior and ventral. This finding aligns with our previous cryosection report (Pattarayingsakul et al. 2019). The continuation of the cuticle-coated structures extending from the lateral wall of the stomach to the early midgut is referred to as the “lateral pyloric valve.” It divides the anterior portion of the midgut into two main chambers: the superior and inferior chambers. Interestingly, the H&E-stained tissue sections show the separation of fine and coarse chymes (fed particles) in the lower and upper chambers, respectively. No previous evidence has reported this phenomenon in shrimps (*Pleoticus muelleri*) (German et al. 2014) and *P. vannamei* (Pattarayingsakul et al. 2019). However, the purpose of this pattern remains unknown. The lateral pyloric valve may serve as a mechanical barrier to prevent coarse particles (indigestible or partially digested food) from mixing with well-digested food in the posterior stomach and anterior midgut. We also observed that the fine chyme, after filtration by the gastric sieve, not only accumulated in the primary and secondary hepatopancreatic spaces but was also found at the intersection area of the hepatopancreas and midgut opening, as well as the proximal part of the anterior midgut (Fig. 3e, f). However, this study could not determine whether the accumulation of fine chyme at the junction and in the early midgut results directly from gastric sieve filtration or the refluxed chyme from the hepatopancreas. To address this issue, tracing feed particles may require further evaluation.

The hepatopancreas, also referred to as the perigastric organ, is reported as a major primary site of infection for the microsporidian *Enterocytozoon hepatopenaei* (EHP) (Tourtip et al. 2009). Oral transmission is a source of disease spread in ponds through cannibalism by infected shrimp (Chaijarasphong et al. 2021). Regarding the route and target tissue of infection, this leads us to focus on EHP infection at the junction of the posterior stomach, hepatopancreas, and anterior midgut. Based on the size of EHP’s spore, approximately 1.1–1.7 × 0.7–1.0 μm (Tourtip et al. 2009; Rajendran et al. 2016), it is quite difficult for it to pass through the gastric sieve, which has a pore size of about 0.2–0.7 µm (Pattarayingsakul et al. 2019). We have previously demonstrated that 0.1-µm fluorescent microbeads can pass through the gastric sieve and enter the hepatopancreas, while 1.0-µm microbeads go directly to the midgut (Pattarayingsakul et al. 2019). In addition, it has been shown that *V. parahaemolyticus* cannot pass through the gastric sieve to enter the hepatopancreas under normal conditions (Li et al. 2025). In this study, the in situ hybridization of EHP transcript detected EHP infection in most of the epithelial cells lining the primary hepatopancreatic space near the junction of the hepatopancreas and anterior midgut (Fig. 7a, b). This apparently suggested that the channel between the hepatopancreas and anterior midgut serves as a potential pathway for EHP infection from the oral route to the hepatopancreas. In addition, EHP infection was also observed in the cell lining of the secondary hepatopancreatic space and the proximal tubules, particularly in B- and R-cells (Fig. 7a, c, d). The absence of an EHP-positive signal in the E-cells at the distal end of the tubule in our study aligns well with reports by Tangprasittipap et al. (2013), Jaroenlak et al. (2016), and in a review by Chaijarasphong et al. (2021). In contrast, Wang et al. (2025) reported the presence of EHP in E-cells using histochemistry. This difference may be due to the varying experimental techniques employed or the severity of the disease in the collected samples.

**Fig. 9:**
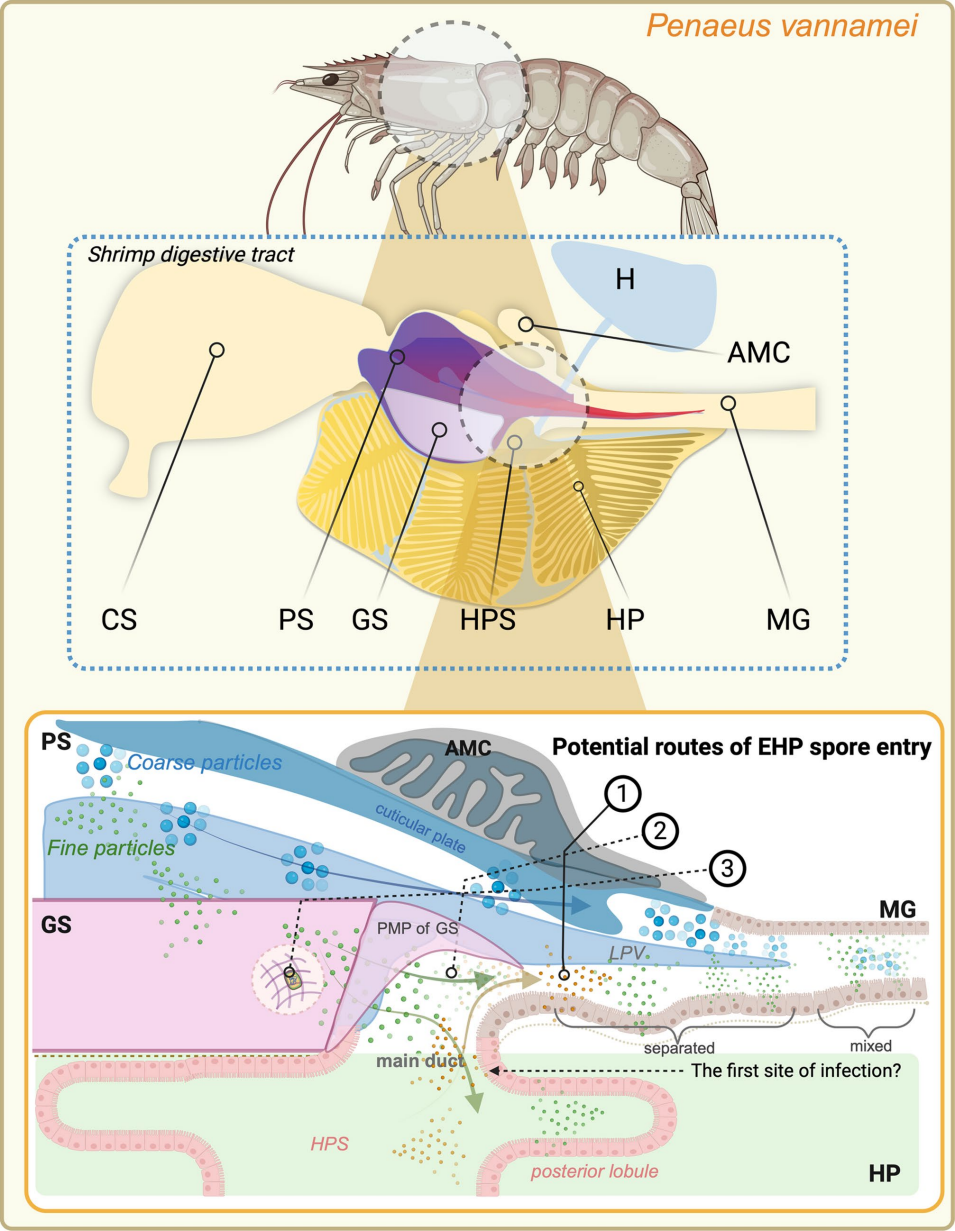
Schematic representation of the digestive structures at the stomach-midgut-hepatopancreatic junction and potential route of EHP spore entry. The lateral pyloric valve (LPV) and the posterior median plate (PMP) of the gastric sieve (GS) constitute a physical barrier that segregates coarse particles (blue arrow) from fine matter. Coarse particles are retained superior to the LPV, while fine particles are filtered through the GS setae into the sub-LPV region, entering either the main hepatopancreatic (HP) duct or the midgut (MG) lumen (green arrows). A secondary communication route below the LPV may facilitate chyme flow between the HP and MG (dark yellow arrow). At the anterior MG, a distinct stratification of digestive content is observed: coarse matter occupies the dorsal portion of the lumen, while fine matter is sequestered ventrally by the LPV before eventually mixing after the LPV terminates. Given that EHP spores (1.1–1.7 × 0.7–1.0 μm in size) might exceed the filtration capacity of the normal-condition GS (0.2–0.7 μm; Pattarayingsakul et al. 2019), we proposed three potential routes of EHP spore entry into the HP: (1) the sub-LPV channel, which provides a direct interface between the HP and MG; (2) the recess between PMP and LPV, which may allow chyme to bypass the GS and connect PS directly to HP/MG transition; (3) the structural compromise or developmental gaps in the GS (e.g., during molting) that impair the filtration efficiency. In all scenarios, the epithelial lining of the HP duct and HPS might represent the initial sites of EHP infection, with subsequent progression to the secondary and tertiary HP ducts. Abbreviation: CS, cardiac stomach; PS, pyloric stomach; H, heart; AMC, anteriormidgutcecum. An illustration was created by BioRender

The gastric sieve has long been considered the gatekeeper of the hepatopancreas. However, the size discrepancy between the GS pores and EHP spores suggests that under normal physiological conditions, the GS setae should exclude EHP spores from the HP. Our discovery of the complex architectural arrangement at the stomach-midgut-hepatopancreas junction (Fig. 9) provides potential routes of EHP spore entry into the HP. This study reveals that the lateral pyloric valves (LPV) and the posterior median plate (PMP) likely serve as chyme-flow regulators. We observed a distinct stratification of chyme within the anterior midgut: coarse particles are confined dorsally, while fine matter is sequestered ventrally by the LPV. We further identified a secondary communication route, a sub-LPV channel, that facilitates a direct interface between the MG and the HPS. This bypass is located posterior to the primary filtration apparatus of the GS. Based on the integration of SR-XTM-CT, histology, and ISH localization, we propose three distinct pathways for EHP internalization (Fig. 9): (1) The sub-LPV channel, which is probably the most likely route for spores that have already entered the midgut. The ventral sequestration of fine chyme by the LPV places spores in immediate proximity to the HP duct orifices. (2) The PMP–LPV recess, which is the structural gap or recess between the PMP and the LPV. This may allow unfiltered chyme to leak directly from the pyloric stomach into the HP transition zone, completely bypassing the GS setae. (3) Physiological windows of vulnerability of the GS. The structural integrity of the GS is not constant. During ecdysis, the shedding of the chitinous foregut lining likely results in a temporary loss of filtration efficiency. This developmental gap may represent a window of opportunity where the HP is essentially unprotected from environmental spores. Our ISH data support this model, showing EHP transcripts mainly in the epithelial lining of primary HP chambers, primary and secondary ducts, and the tertiary duct opening. This suggests these areas are initial infection sites.

## Conclusion

This study provides a high-resolution, three-dimensional characterization of the pyloric-midgut-hepatopancreas junction in the white shrimp, *Penaeus vannamei*, through the synergistic application of SR-XTM µ-CT, histology, and SEM. The volumetric visualization provided by SR-XTM µ-CT offers novel insights into the mechanical complexities of the crustacean digestive transport system and highlights potential bypass routes for pathogen internalization, particularly for EHP. As molecular and omics-based approaches increasingly dominate crustacean research, these high-resolution architectural data provide a critical structural framework for the spatial mapping and functional interpretation of molecular localization within the digestive landscape.

## Supplementary Information

Below is the link to the electronic supplementary material.

ESM1 (DOCX 5.08 MB)

## Data Availability

All data are available in the main text and the supplements.

## References

[CR1] Bell TA, Lightner DV (1988) A handbook of normal penaeid shrimp histology. World Aquaculture Society, Baton Rouge, Louisiana

[CR2] Caceci T, Neck KF, Lewis DDH, Sis RF (1988) Ultrastructure of the hepatopancreas of the Pacific white shrimp, *Penaeus vannamei* (Crustacea: Decapoda). J Mar Biol Assoc U K 68:323–337. 10.1017/S002531540005222X

[CR3] Ceccaldi H (1989) Anatomy and physiology of digestive tract of crustaceans decapods reared in aquaculture. Actes de Colloques - IFREMER, (9), 243–259. https://archimer.ifremer.fr/doc/00000/1486/

[CR4] Chaijarasphong T, Munkongwongsiri N, Stentiford GD et al (2021) The shrimp microsporidian *Enterocytozoon hepatopenaei* (EHP): biology, pathology, diagnostics and control. J Invertebr Pathol 186:107458. 10.1016/j.jip.2020.107458 32882232 10.1016/j.jip.2020.107458

[CR5] Chapuis L, Andres C-S, Gerneke DA, Radford CA (2024) Bioimaging marine crustacean brain: quantitative comparison of micro-CT preparations in an Alpheid snapping shrimp. Front Neurosci 18:1428825. 10.3389/fnins.2024.1428825 39659887 10.3389/fnins.2024.1428825 PMC11628493

[CR6] Desrues J, Viggiani G, Bsuelle P (Eds.) (2006) Advances in X-ray Tomography for Geomaterials. ISTE. 10.1002/9780470612187

[CR7] Ding Y, Vanselow DJ, Yakovlev MA et al (2019) Computational 3D histological phenotyping of whole zebrafish by X-ray histotomography. Elife 8:e44898. 10.7554/eLife.44898 31063133 10.7554/eLife.44898 PMC6559789

[CR8] German DP, Gawlicka AK, Horn MH (2014) Evolution of ontogenetic dietary shifts and associated gut features in prickleback fishes (Teleostei: Stichaeidae). Comp Biochem Physiol B Biochem Mol Biol 168:12–18. 10.1016/j.cbpb.2013.11.006 24269211 10.1016/j.cbpb.2013.11.006

[CR9] Jaroenlak P, Sanguanrut P, Williams BAP et al (2016) A nested PCR assay to avoid false positive detection of the microsporidian *Enterocytozoon hepatopenaei* (EHP) in environmental samples in shrimp farms. PLoS One 11:e0166320. 10.1371/journal.pone.0166320 27832178 10.1371/journal.pone.0166320 PMC5104377

[CR10] Kasamechotchung C, Munkongwongsiri N, Plaipetch P et al (2025) Effect of partial and total replacement of fishmeal by soybean meal in feed on growth and gut performance of *Penaeus vannamei*. Sci Rep 15:451. 10.1038/s41598-024-83494-1 39747937 10.1038/s41598-024-83494-1 PMC11697264

[CR11] Li L, Wang Y, Jin L et al (2025) Effects of hypoxic stress on gastric sieve (GS) filtering function of *Litopenaeus vannamei*. Aquaculture 599:742169. 10.1016/j.aquaculture.2025.742169

[CR12] Limaye A (2012) Drishti: a volume exploration and presentation tool. In: Stock SR (ed) San Diego. California, USA, p 85060X

[CR13] Martín-Vega D, Garbout A, Ahmed F et al (2018) 3D virtual histology at the host/parasite interface: visualisation of the master manipulator, *Dicrocoelium dendriticum*, in the brain of its ant host. Sci Rep 8:8587. 10.1038/s41598-018-26977-2 29872086 10.1038/s41598-018-26977-2 PMC5988677

[CR14] Meth R, Wittfoth C, Harzsch S (2017) Brain architecture of the Pacific white shrimp *Penaeus vannamei* Boone, 1931 (Malacostraca, Dendrobranchiata): correspondence of brain structure and sensory input? Cell Tissue Res 369:255–271. 10.1007/s00441-017-2607-y 28389816 10.1007/s00441-017-2607-y

[CR15] Metscher BD (2009) MicroCT for comparative morphology: simple staining methods allow high-contrast 3D imaging of diverse non-mineralized animal tissues. BMC Physiol 9:11. 10.1186/1472-6793-9-11 19545439 10.1186/1472-6793-9-11 PMC2717911

[CR16] Munkongwongsiri N, Aldama-Cano DJ, Suebsing R et al (2021) Microsporidian *Enterocytozoon hepatopenaei* (EHP) spores are inactivated in 1 min at 75 °C. Aquaculture 533:736178. 10.1016/j.aquaculture.2020.736178

[CR17] Pattarayingsakul W, Pudgerd A, Munkongwongsiri N et al (2019) The gastric sieve of penaeid shrimp species is a sub-micron nutrient filter. J Exp Biol jeb.199638. 10.1242/jeb.199638 10.1242/jeb.199638 31028105

[CR18] Rajendran KV, Shivam S, Ezhil Praveena P et al (2016) Emergence of *Enterocytozoon hepatopenaei* (EHP) in farmed *Penaeus* (*Litopenaeus*) *vannamei* in India. Aquaculture 454:272–280. 10.1016/j.aquaculture.2015.12.034

[CR19] Rivera-Quiroz FA, Miller JA (2022) Micro-CT visualization of the CNS: performance of different contrast-enhancing techniques for documenting the spider brain. J of Comparative Neurology 530:2474–2485. 10.1002/cne.25343 10.1002/cne.25343 PMC9540357 35598086

[CR20] Roberts NL (1966) Morphology and Histology of the Stomach of the White Shrimp Penaeus fluviatilis (Say,1817). Dissertations. 2042. https://aquila.usm.edu/dissertations/2042

[CR21] Tang KFJ, Han JE, Aranguren LF et al (2016) Dense populations of the microsporidian *Enterocytozoon hepatopenaei* (EHP) in feces of *Penaeus vannamei* exhibiting white feces syndrome and pathways of their transmission to healthy shrimp. J Invertebr Pathol 140:1–7. 10.1016/j.jip.2016.08.004 27530403 10.1016/j.jip.2016.08.004

[CR22] Tangprasittipap A, Srisala J, Chouwdee S et al (2013) The microsporidian *Enterocytozoon hepatopenaei* is not the cause of white feces syndrome in whiteleg shrimp *Penaeus* (*Litopenaeus*) *vannamei*. BMC Vet Res 9:139. 10.1186/1746-6148-9-139 23856195 10.1186/1746-6148-9-139 PMC3717009

[CR23] Tourtip S, Wongtripop S, Stentiford GD et al (2009) *Enterocytozoon hepatopenaei* sp. nov. (Microsporida: Enterocytozoonidae), a parasite of the black tiger shrimp *Penaeus monodon* (Decapoda: Penaeidae): fine structure and phylogenetic relationships. J Invertebr Pathol 102:21–29. 10.1016/j.jip.2009.06.004 19527727 10.1016/j.jip.2009.06.004

[CR24] Wang Y, Lin S, Zhao M et al (2025) Tissue and cell types infected by *Ecytonucleospora hepatopenaei* (EHP). J Invertebr Pathol 211:108344. 10.1016/j.jip.2025.108344 40294744 10.1016/j.jip.2025.108344

[CR25] Young JH (1959) Morphology of the white shrimp, *Penaeus setiferus*, (Linnaeus 1758). Fish Bull 59:1–168

[CR26] Zhu B, Wang Z, Li Y et al (2024) Morphological and structural analysis of *Penaeus vannamei* mandibles and an attempt at real-time cannibalism monitoring based on passive acoustics. Aquac Rep 37:102199. 10.1016/j.aqrep.2024.102199

