# Supplementary material for: Unlocking the hidden anatomy: synchrotron micro-tomography of the stomach, midgut, and organs in Penaeus vannamei and the potential route of Enterocytozoon hepatopaenaei (EHP) infection
Source: Cell Tissue Res. 2026 Apr 24;404(2):7. doi: 10.1007/s00441-026-04067-4 (PMC13106256; doi:10.1007/s00441-026-04067-4)
Supplement: Supplementary file 1 — (DOCX 5.08 MB) [file 441_2026_4067_MOESM1_ESM.docx]

**Unlocking the Hidden Anatomy: Synchrotron Micro-Tomography of the Stomach, Midgut, and Organs in Penaeus vannamei and the Potential Route of**

**Enterocytozoon hepatopaenaei (EHP) Infection**

Thanapong Kruangkum^1,2^, Phakkhananan Pakawanit^3^, Kornchanok Jaiboon^1,2^, Piyachat Sanguanrut^4^, Sukanya Saedan^1,2^, Kallaya Sritunyalucksana^4^, Siripong Thitamadee^2,5,6^, Rapeepun Vanichviriyakit^1,2*^

**Supplementary Data**

**
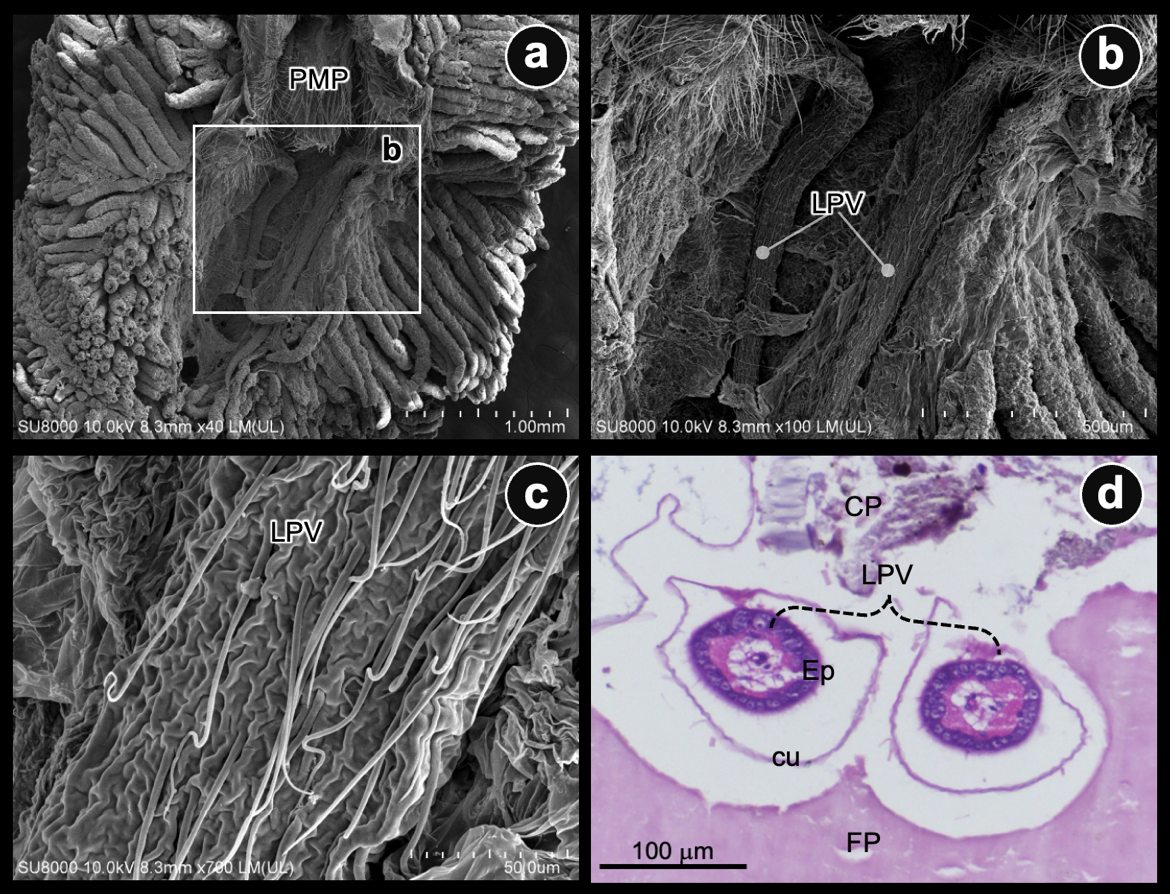
**

**Figure S1:** The SEM and H&E photographs of the lateral pyloric valves (LPV). (a) Low and (b) medium magnified photographs of the LPV at the posterior portion of the pyloric stomach by SEM. (c) SEM high magnification of the LPV revealed the cuticle-coated structure with a large number of hook-like setae. (d) The histology of LPV consists of epithelium (Ep) and the core connective tissue, which is covered by a cuticle (cu). *Abbreviation: CP, crude particle; FP, fine particles.*


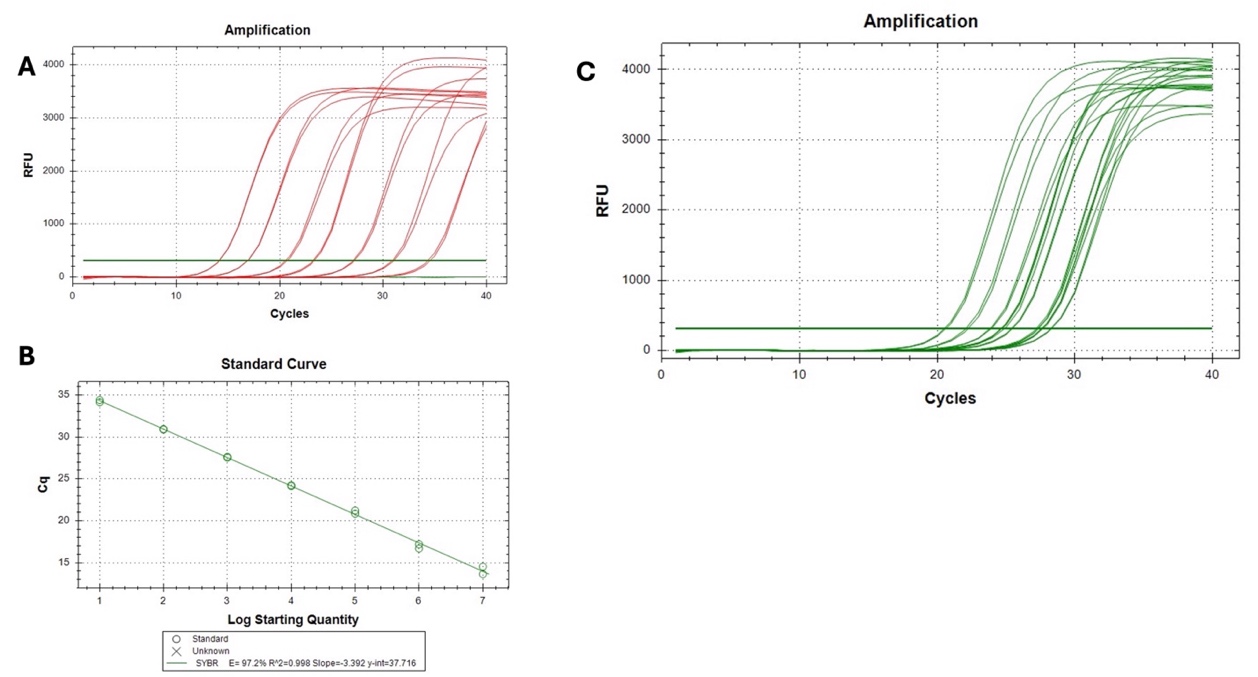


**Figure S2.** Real-time PCR detection of EHP spore wall gene using the SYBR Green-qPCR method. (A) Amplification plot and (B) standard curve for determining EHP copy numbers. (C) Amplification plot of 10 shrimp samples showing EHP detected in all samples.

**
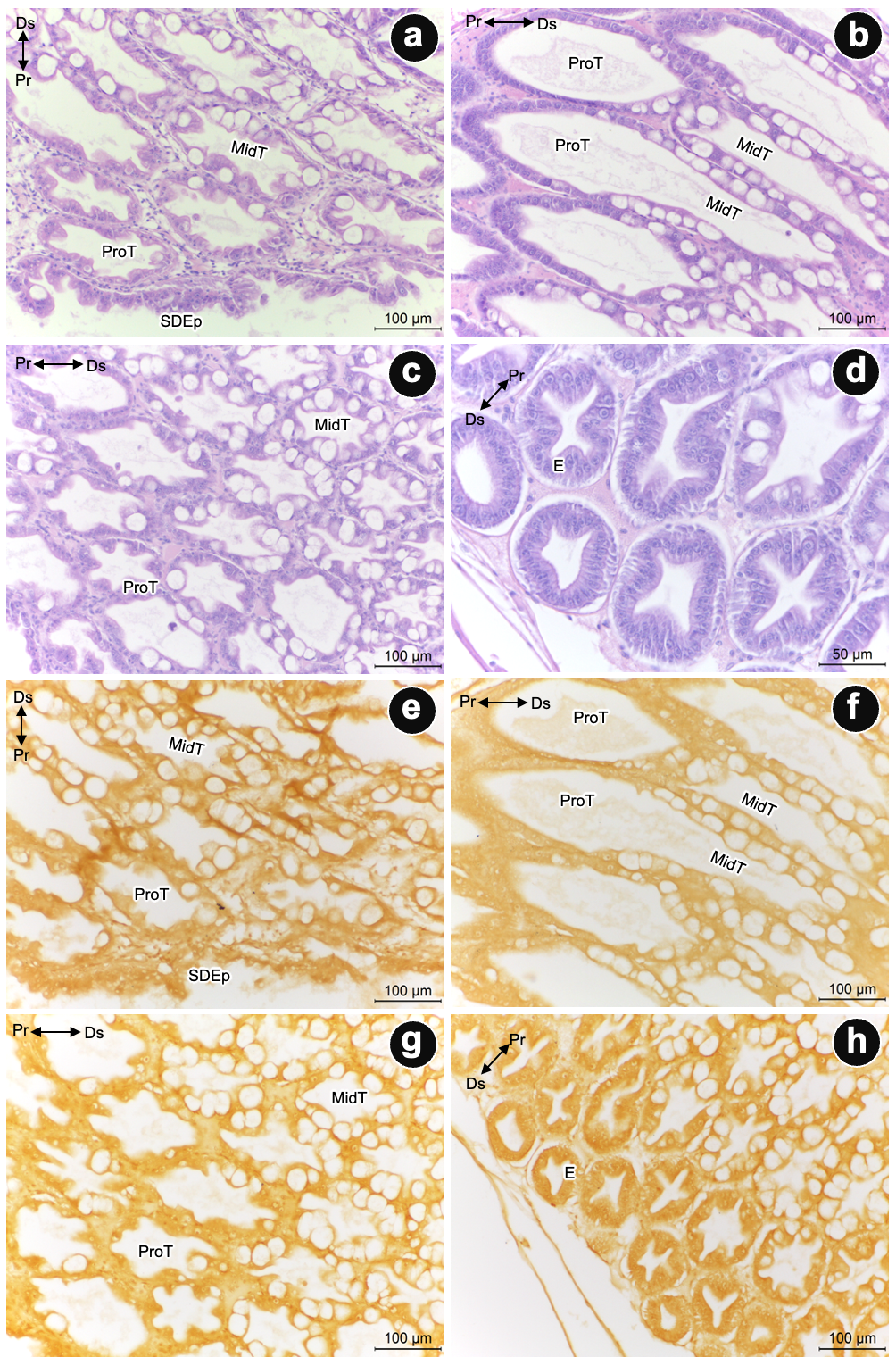
**

**Figure S3:** The photographs of H&E staining and negative control without probe for in situ hybridization.

(a-d) The H&E-stained HP tissue of EHP-infected shrimp and (e-h) negative control without probe incubation, which are presented corresponding to the same location of positive tissue sections. Note that this is the early phase of EHP infection; the typical signs of EHP infection, such as spores and epithelial sloughing, are not present. Depletion of lipid vacuoles in the R-cells is observed. *Abbreviations: Pr, proximal; Ds, distal; MidT, middle tubule; ProT, proximal tubule; SDEp, secondary duct epithelium; E, E-cell.*

*
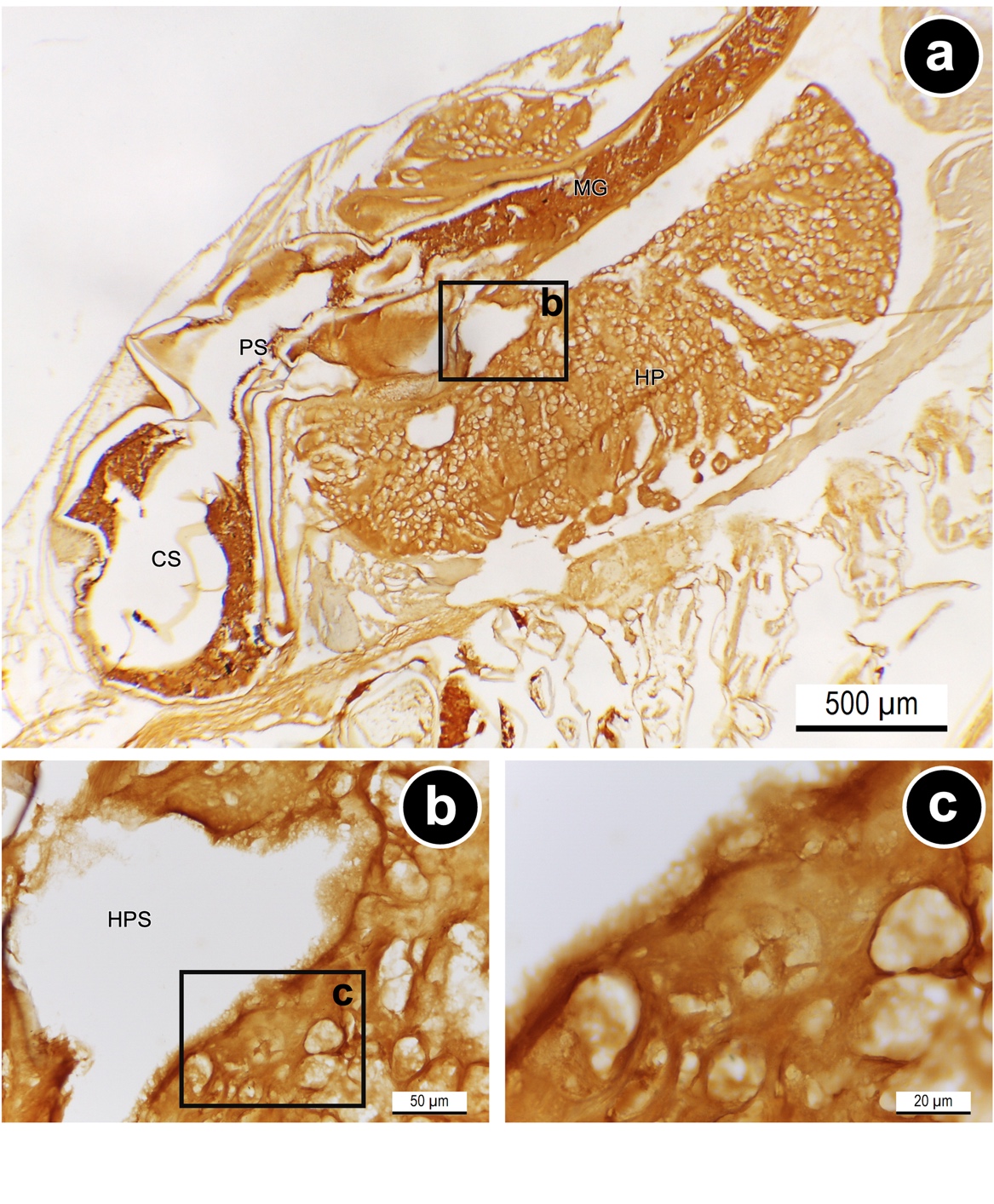
*

**Figure S4:** Photographs of non-infected tissue after probe treatment for in situ hybridization. The results show only background staining. *Abbreviations: CS, cardiac stomach; PS, pyloric stomach; HP, hepatopancreas; MG, midgut; HPS, hepatopancreatic space.*
